# The Development and Implementation of Airflow Visualization Studies (“Smoke” Studies) as a Training Tool in Aseptic Hospital Compounding Facilities

**DOI:** 10.3390/pharmacy10050101

**Published:** 2022-08-23

**Authors:** Paula E. Borgonje, Lisa Wibier, Patrick Noordman, Herman J. Woerdenbag, Bahez Gareb

**Affiliations:** 1 Department of Clinical Pharmacy, Meander Medical Centre, Maatweg 3, 3813 TZ Amersfoort, The Netherlands; 2 Apotheek A15, Buys Ballotstraat 2, 4207 HT Gorinchem, The Netherlands; 3 Department of Clinical Pharmacy and Toxicology, Martini Hospital Groningen, Van Swietenplein 1, 9728 NT Groningen, The Netherlands; 4 Department of Pharmaceutical Technology and Biopharmacy, University of Groningen, Antonius Deusinglaan 1, 9713 AV Groningen, The Netherlands; 5 Department of Clinical Pharmacy and Pharmacology, University Medical Center Groningen, Hanzeplein 1, 9713 GZ Groningen, The Netherlands

**Keywords:** smoke studies, airflow visualization, aseptic handling, LAF cabinet, training, qualification, good manufacturing practice, GMP

## Abstract

In the compounding facilities of hospital pharmacies, extemporaneous preparations for parenteral administration are produced using aseptic handling. The designated environment for this practice is a clean area, such as a laminar airflow (LAF) cabinet placed in a classified cleanroom complying with good manufacturing practices (GMP) and International Organization for Standardization (ISO) 14644-1 guidelines. The European GMP Annex 1 (Revision 2020) and United States Pharmacopeia (USP) <797> monograph state that airflow visualization studies (“smoke” studies) should be performed to substantiate the cleanroom and LAF cabinet performance and their qualification status. Even though smoke studies are required by these guidelines, current literature does not describe detailed practical protocols and acceptance criteria. The objective of this study was to develop and implement a practical smoke study protocol to ensure compliance with aseptic handling guidelines in hospital pharmacies. First, a literature search was performed to collect information about smoke study protocols and acceptance criteria. Subsequently, a smoke study protocol was developed for a downflow and crossflow LAF cabinet as well as for grade C/B cleanroom areas. As a proof of concept, the smoke study protocol for the downflow LAF cabinet was executed in the at-rest and in-operation states. Video recordings of the smoke studies were analyzed to assess the performance of the cabinet. Finally, the video recordings obtained from the smoke studies were used in a training program for hospital pharmacy operators, which showed that smoke studies might aid in operators’ aseptic handling awareness. To the best of our knowledge, the present study provides for the first time a practical approach for the development of smoke study protocols in a hospital pharmacy setting and shows potential for training operators, process optimization, and continuous quality improvement.

## 1. Introduction

Compounding facilities in hospital pharmacies are important for providing patients with individualized treatment in the form of extemporaneous preparations. Many of these preparations are sterile products for parenteral administration and produced using aseptic handling. When applying this technique, terminally sterilized drug products, containers, and ancillary devices are used to compound sterile extemporaneous preparations such as infusions or injections for individual patients. In general, this is performed aseptically in designated clean areas (the background environment) in which a laminar air flow (LAF) cabinet is situated. The clean areas usually are cleanrooms complying with good manufacturing practice (GMP) and NEN-EN-ISO 14644-1 guidelines [1,2]. Depending on the country and continent where the hospital pharmacy is located, applicable guidelines concerning aseptic preparations are GMP Annex 1 [1], Ph. Eur. 2619 [2], or USP <797> [3].

The GMP Annex 1 (Revision 2020) and USP <797> monographs require that airflow visualization tests be carried out to substantiate the cleanroom and LAF cabinet performance and qualification status. For instance, the GMP Annex 1 revision states: “The airflow pattern studies should be performed both at rest and in operation. Video recordings of the airflow patterns should be retained. The outcome of the air visualization studies should be considered when establishing the facilities environmental monitoring program” [1].

Airflow visualization studies, or “smoke” studies, are tests in which smoke-generating devices are placed in various spots in designated areas. The visualized airstreams are recorded and analyzed with the objective of assessing whether the airflow patterns are appropriate [4]. For instance, turbulence or unidirectional airflow disruption caused by incorrect object placement or uncontrolled movements of personnel can be detected. Such observations may be used for process optimization and training personnel [5,6]. Smoke studies are relatively cheap and easy to perform and can quickly help to discover potential airflow disruption (e.g., due to improper handling) or air leaks. However, objective interpretations of the results can be challenging, and it is therefore recommended that multiple observers analyze the results for a reliable interpretation.

Interestingly enough, even though conducting smoke studies is generally required, there is no definite and practical guideline for these airflow visualization tests. For instance, a literature search in, among others, PubMed, Web of Science, the European and British Pharmacopoeia, and the United States Pharmacopeia as well as the European and American GMP guidelines did not reveal any practical information or details with regards to these tests [1,3,5,6,7]. In addition, no Parental Drug Association (PDA) technical report is available on this subject [8]. Moreover, hospital pharmacies differ from the pharmaceutical industry in several ways. Typically, hospital pharmacies have fewer resources, smaller production facilities and batch sizes, but larger product ranges than a given pharmaceutical company. Consequently, hospital pharmacy operators are trained for a wide range of compounding activities that take place in LAF cabinets. In addition, apparatus, tools and utensils are often placed in a LAF cabinet to aid in compounding. This emphasizes the importance of a thorough understanding of airflow patterns and influencers thereof in LAF cabinets. Training methods that facilitate in this understanding are therefore relevant. They are needed for a good understanding of airflow patterns, proper aseptic handling, and process optimization.

The objective of this study was to develop and implement smoke studies in the hospital pharmacy setting. Additionally, the results obtained from the smoke studies were used in a training program for hospital pharmacy compounding personnel in view of process optimization and continuous quality improvement.

## 2. Materials and Methods

### 2.1. Development of the Smoke Study Protocol

The study was performed in the compounding facility of the Martini Hospital (Groningen, The Netherlands). In anticipation of the implementation of EU GMP Annex 1 revision 2020, a search for regulatory requirements and practical guidelines for the development and implementation of airflow visualization studies was performed within the GMP, Ph. Eur. 2619, USP <797>, NEN-EN-ISO 14644-1, ICH guidelines, PIC/S guides, and PDA technical reports [1,3,5,8,9,10,11]. Additionally, a search for practical guidelines or considerations for the development and implementation of these studies was performed within Web of Science and Pubmed Library. Various search queries were created to obtain appropriate articles or books. The search queries contained one or more of the following terms: smoke, smoke test, fog, airflow, visualization, laminar, unidirectional, cleanroom, isolator and cabinet. A Google search was performed to find suppliers of smoke guns. In addition, footage of smoke studies on video platforms and search engines including YouTube and Google was obtained.

No practical guideline or acceptance criteria were found with the search strategy. Therefore, we developed a practical approach for the development and implementation of smoke study protocols. This protocol and the acceptance criteria are given in Section 3. As a proof of concept, the downflow LAF cabinet smoke study protocol was executed (Section 2.2). The results obtained from the smoke study protocol were used to evaluate the performance of the downflow LAF cabinet (at rest and in operation) and to develop the training program for hospital pharmacy operators (Section 2.3).

### 2.2. Smoke Studies

Smoke studies were performed using a Dräger FlowCheck (Dräger, Lübeck, Germany) smoke gun. This device generated smoke based on glycerin. The smoke gun was placed in various locations according to the developed protocol in a Holten Maxi Safe Model 1.2 (Thermo Fisher Scientific Inc., Waltham, MA, USA) downflow LAF cabinet (depth × width × height: 58 × 120 × 70 cm), which was located in a controlled room dedicated to aseptic handling that had no GMP classification. Studies were performed in the downflow LAF cabinet both at rest and during compounding. Aseptic process simulation consisted of taking 10 mL of water for injections (WFI) from a 100 mL container, reconstituting powdered concentrated tryptic soya broth (TSB) (InstaMediA, BioTrading, Mijdrecht, The Netherlands) with this, and adding the concentrated TSB to a 250 mL NaCl 0.9% infusion bag. The smoke streams were recorded on a tripod, and the footage was analyzed by inspecting subsequent frames of the video.

### 2.3. Training Program

Based on the obtained smoke study results, a 1.5 h LAF training program was developed for the hospital pharmacy operators. The following items were incorporated into the training program: contamination risks during aseptic handling, differences between unidirectional and turbulent airstreams, airstreams in a LAF cabinet (crossflow/downflow), and the possible effects of disturbing the laminar airflow on the quality of aseptic compounding.

Before and after attending the training program, the personnel filled out a questionnaire about aseptic compounding and airflows in cleanrooms and LAF cabinets. The questionnaire comprised (1) amount of operator experience with regard to working in LAF cabinets, (2) knowledge of aseptic compounding and airflows, and (3) self-evaluation. The numbers of correct answers in part (2) were compared before and after the training program using a one-sided paired-samples *t*-test (*p* value < 0.05). After the training, participants were also asked their opinion (reflection) of the added value of smoke studies as a training tool for new employees. The questionnaire can be found in Supplementary Document S1.

## 3. Results and Discussion

### 3.1. Development of the Smoke Study Protocol

The results from the literature study showed that the selection of a suitable smoke generator for the desired use is essential. The smoke generator must produce a sufficient amount of smoke to adequately visualize air streams. The smoke must be nontoxic, easy to clean, and light and ideally have a low bioburden [12]. The NEN-EN-ISO 14664 part 3 mentions that a smoke generator should release droplets with a size of 1 to 10 µm. The particle generation rate should be 1 to 25 g/min [9]. Water-based smoke is preferred over oil-based smoke because it disperses more quickly and is easier to clean, although it is more difficult to capture on camera [13].

In general, there are three types of commercially available smoke machines that can be used in cleanrooms. Traditional smoke machines are larger, non-portable machines with separate nozzles that must be attached to the machine. Portable smoke machines are more compact but still generate a considerable amount of smoke. Portable smoke guns are the smallest smoke machines and are ideal for visualizing the airflow in a LAF cabinet or in hard-to-reach areas. Our search strategy resulted in a list of commercially available smoke machines that are suitable for smoke studies in cleanroom areas (Supplementary Table S1). This list may aid hospital pharmacies in their choice of the desired smoke machine.

The airflow visualization video recordings are the data of interest from the smoke studies. These recordings should therefore be treated as any other data generated on site and should comply with GMP data integrity procedures [14]. The positions and lighting for the video recording must be determined beforehand, and the camera operator should be appropriately trained and instructed to be able to record the data with sufficient contrast between the smoke and the background. It might be beneficial to use two cameras to capture the airflow visualization from multiple angles during a given test. The footage should be edited as little as possible, since bias could be introduced and important information could potentially be lost.

Aside from the video recordings, a written summary report (based on critical and continuous observation) could outline any airflow disruption or other shortcomings regarding airflow or aseptic handling. The airflow should be described as clearly as possible using predefined terms such as smooth, flowing, turbulent, unidirectional, or sweeping [14]. Adding relevant (representative) images to the protocol supports the observer when drafting the report. Finally, predefined acceptance criteria should be clear and in place since only then it can be objectively concluded whether the setup passes or fails the test.

Considering all of the above, we developed a practical smoke study protocol that is given in Table 1. The tests were designed in such a way that instructed pharmacy operators could easily perform the tests. The acceptance criteria were chosen based on critical attributes and the desired performance of the cabinets and cleanrooms.

For our assessment of airflow in the downflow LAF cabinet, a portable smoke gun was the preferred choice since it was easy to handle and could be placed in different positions within the cabinet, the smoke in the cabinet could be visualized from multiple angles, and the device was relatively cheap. However, a disadvantage of the smoke gun was the generation of smoke based on glycerin, which leaves a residue and requires extra-thorough cleaning and disinfection steps. Compared with larger and more expensive smoke machines, the portable smoke gun generates a less-dense smoke, making it less suitable for analyzing the airflows in bigger areas such as grade B and C cleanrooms. Therefore, we advise choosing the type of smoke machine based on the intended purpose of the smoke studies.

### 3.2. Smoke Studies

**Figure 1:**
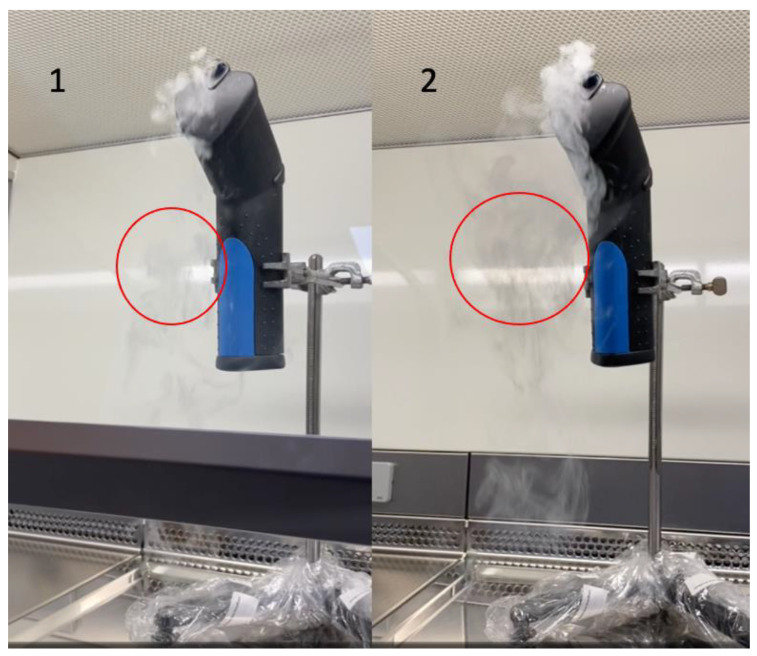
The equipment used during the downflow LAF cabinet smoke study protocol. Image (**1**) and (**2**) show representative images during the visualization of vertical airflow test. (**1**): Sash closed. (**2**): Sash opened.

The smoke studies were performed in a downflow LAF cabinet with the smoke study protocol described in Table 1**.** Figure 1 shows the equipment that was used during these tests. The smoke gun was placed in a stainless steel adjustable holder that was easy to disinfect and could be used to place the smoke gun in any location inside the LAF cabinet. In the position shown in Figure 1, there was the least disruption of laminar airflow by the used equipment. The airflow could be accurately visualized, and disruptions of the unidirectional flow could be identified.

The potential risk of subjectivity in the interpretation of the result was considered a pitfall of the smoke studies. The position of the camera, as well as the lighting conditions, has a substantial effect on the visibility of the airflow. Furthermore, the background of the used smoke study equipment is of importance for the visualization of the smoke stream. For instance, it may be challenging to observe the white smoke against a white/light background. However, it is not always possible to change the background color of the footage, especially in LAF cabinets. The introduction of foreign-colored sheets may not only affect the investigated airflow but may also be undesirable in view of contamination control.

Taken together, we recommend predefining the best filming positions based on preliminary smoke tests that are conducted with the equipment as it is intended to be used during the smoke study protocol. In addition, not only should the acceptance criteria investigate critical attributes, it should also be considered whether the criteria can be adequately and reproducibly visualized with the smoke tests. Moreover, it is advisable that two operators be present during the studies and assess the test results independently, which is in line with the four-eye principle of the interpretation of GMP results.

All the test results for the downflow LAF cabinet complied with the listed acceptance criteria (Table 1). The vertical airstreams started to deflect into horizontal airstreams 25–30 cm above the work surface of the cabinet. Smoke did not escape from the cabinet, even when the sash was fully opened. The red circles in Figure 1 show that the smoke spreads out more when the sash is open (Figure 1(2) than when the sash is closed (Figure 1(1)). Opening the sash did thus cause a slight turbulence. Smoke that was generated outside the cabinet, up to a distance of 20–25 cm, was drawn into the front vent and did not enter the clean working area of the cabinet.

Smoke studies were also performed during simulated operations in the LAF cabinet. The results showed that airstreams quickly re-established a unidirectional nature after being disturbed by objects or personnel. The placement of large objects caused upward airstreams but no observed turbulence (Supplementary Video S1). Vigorously opening and closing the door of the background area did not affect the airflow. Walking near the front of the cabinet caused a slight deflection that increased when the sash was fully opened (Supplementary Video S2).

**Figure 2:**
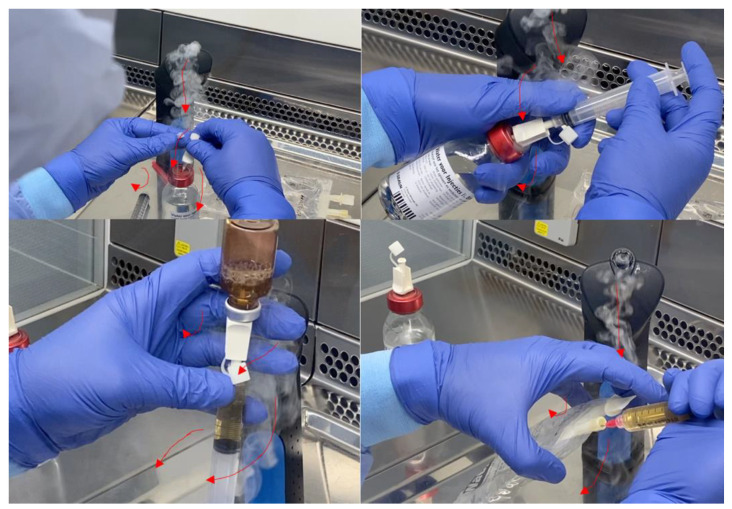
Representative images of the aseptic process simulation, incorrect method. The images show first air blockage of critical points (e.g., sterile septum), which is considered a contamination risk factor [4]. The red arrows indicate the direction of the airflow.

**Figure 3:**
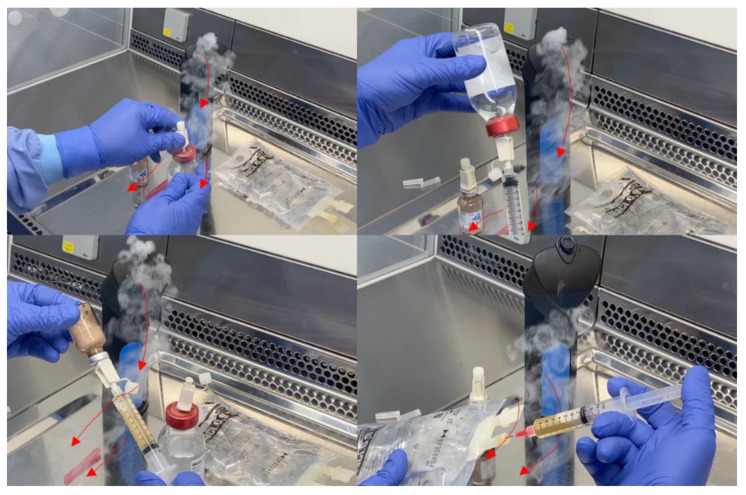
Representative images of the aseptic process simulation, correct method. The images show that first air blockage of critical points (e.g., sterile septum) is avoided, which is desirable during aseptic handling [4]. The red arrows indicate the direction of the airflow.

An aseptic process simulation was performed, which consisted of reconstituting a dry-powder formulation and subsequently withdrawing and adding the solution to a sodium chloride infusion bag with a syringe. Hand movements caused small areas of turbulence under the hands. When the operator placed their hands nearby critical points, “first air” (i.e., particle-free air exiting the HEPA filter in a unidirectional air stream [4]) was blocked, and turbulences underneath the palm were observed (Figure 2). By carefully placing syringes and vials in skewed positions and keeping hands away from critical points, first air blockage was avoided (Figure 3).

All movements and the placement of products influenced the airflow in the downflow LAF cabinet. During aseptic handling, the blockage of first air near critical points poses a risk of contamination that should be taken into account [16]. For example, the smoke study results from an aseptic process simulation using a BAXA repeater pump in a downflow LAF cabinet showed that it is practically impossible to execute the compounding process without first air blockage [16]. This suggests that this process is less suitable for a downflow compared with a crossflow LAF cabinet. Performing smoke studies during aseptic process simulations makes it possible to visualize such blockages and aid in process optimization and validation. The objective of the tests will be process specific (e.g., avoiding the blockage of first air around critical points). Therefore, special attention should be paid to the acceptance criteria when apparatus, utentils, or other tools are used. Footage showing undesired situations as opposed to correct aseptic handling may be used to train pharmacy operators.

### 3.3. Training Program

A total of 16 hospital pharmacy operators followed the training program, and 6 operators with mean experience in aseptic handling of 7.4 years filled out the questionnaire both before and after the training program. They produced on average 2–3 aseptically manufactured batches per week, which consisted of 100–300 units per batch. On the knowledge part of the questionnaire, the respondents scored 9.3 points before the training and 12.0 points after the training (*p* < 0.05). This should be interpreted with the understanding that the same questions were used before and after the training program.

Before the training program, operators stated that they were conscious about the airflow patterns when placing objects into LAF cabinets. Furthermore, they stated that they were conscious about moving slowly and carefully in and around the cabinet. After the training program, operators stated that the training was informative and helpful. In addition, they named more possible ways to improve aseptic handling techniques than before the training, such as avoiding overcrowding the cabinet, working more calmly, and moving less near the cabinet. The operators were positive about the training program and considered it a useful tool for training new operators as well as for increasing the awareness of experienced operators. Even though the analysis of the training program was limited by the small number of participants, the results were considered indicative of the educative potential of the smoke studies and training program. 

## 4. Conclusions

In conclusion, even though conducting smoke studies is generally required, the current literature does not describe a definite and practical guideline for these airflow visualization tests. Based on the outcome of our studies, we are able to supplement existing information regarding airflow visualization tests with concrete procedures and criteria. Furthermore, we showed that smoke studies provide a useful tool for training in aseptic handling.

## Figures and Tables

**Table 1:** Recommendations for airflow visualization tests. Unless otherwise specified, all tests should be performed with the LAF cabinet turned on and the sash at working height.

Location	State	Test	Description	Acceptance Criteria
**Downflow LAF cabinet**	At rest	Visualization of vertical airflow	Supply smoke at the middle depth and middle height at three points evenly spaced across the width. Perform with sash opened and sash at working height.	Unidirectional, no turbulence, no smoke escaping from the cabinet, similar at all tested positions [14].
		Downflow Test	Passing smoke from one end of the cabinet to the other along the center line at 15 cm above the top of the access opening [15].	Unidirectional at all points, no turbulence. No dead spots or reflux [15].
		View Screen Retention Test	Passing smoke from one end of the cabinet to the other 2.5 cm behind the sash at 15 cm above the top of the access opening [15].	Unidirectional at all points, no dead spots or reflux, no smoke escaping from the cabinet [15].
		Work Opening Edge Retention Test	Passing smoke around the edges of the sash opening at 2.5 cm outside the cabinet, with particular attention paid to corners and vertical edges [15].	No smoke refluxes out of the cabinet once drawn in, nor does smoke billow over the work surface or penetrate onto it [15].
		Sash Seal Test	Passing smoke up the inside of the window at the side channel seals [15].	Unidirectional at all points, no smoke escaping from the cabinet [15].
		Airflow near the vents ^1^	Passing smoke from one end of the cabinet to the other, at 10 cm height and 5–7 cm distance from the back vent as well as from the front vent.	All smoke is drawn into the vents, no turbulence.
		Deflection of vertical airflow	At three points evenly spaced across the width and middle depth, pass smoke from the top of the cabinet to the bottom.	Unidirectional at all points. This test is useful to visualize the point of deflection.
		Airflow outside of the cabinet	At three points in height (height of the sash, working height and the middle of these heights), pass smoke from one end of the cabinet to the other, at increasing distance from the cabinet.	No smoke enters the cabinet, all smoke is eliminated by the front vents. This test is useful to visualize up to which distance the air is influenced by the suction of the vent.
	In operation	Hand movements in the cabinet	Supplying smoke from above the working area. Placing two hands under the smoke supply, in the middle of the working area.	Smoke moves over and around hands and unidirectional flow re-establishes. No turbulence.
		Opening door	Vigorously opening the door of the operating room, while visualizing vertical airflow (see “Visualization of vertical airflow”).	See “Visualization of vertical airflow”. Opening the door of the operating room does not influence airflow in the cabinet.
		Slow movements around cabinet	Walking past the cabinet slowly, while visualizing vertical airflow (see “Visualization of vertical airflow”).	See “Visualization of vertical airflow”. Walking past the cabinet does not influence airflow in the cabinet.
		Fast movements around cabinet	Walking past the cabinet quickly, while visualizing vertical airflow (see “Visualization of vertical airflow”).	See “Visualization of vertical airflow”. If any disturbances, airflow should re-establish immediately. This test is useful to visualize influence of fast movements around the cabinet.
		Placing vials and flasks	Placing routinely used vials of different sizes and shapes at several places in the working field.	Airflow is unidirectional and moves around the vial. If airflow is disturbed by placing objects in the cabinet, it re-establishes immediately.
		Aseptic process simulation	Perform an aseptic process simulation while supplying smoke over the working field (e.g., reconstitute powder in a vial and inject this into an infusion bag) [16].	No air escapes from the cabinet while compounding. First air^2^ should never be blocked near a critical spot [16]. This test is useful to visualize the effect of an operator’s actions.
**Crossflow LAF cabinet**	At rest	Visualizing horizontal airflow	Passing smoke along the HEPA-filter on the back panel, 10–20 cm away from the filter). Move the smoke machine from left to right, top to bottom.	The airflow patterns are unidirectional at rest: the air sweeps away from the HEPA-filter. There is no noticeable turbulence or disruption of the unidirectional airflow [14].
		Airflow outside the cabinet	Passing smoke along the cabinet (left to right). Position the smoke machine 1.5 cm outside of the cabinet.	Air from outside the cabinet is not drawn into the crossflow cabinet.
	In operation	Hand movements in the cabinet	Supplying smoke from behind the working area. Placing two hands in the smoke supply, in the middle of the working area.	Smoke moves around hands and unidirectional flow re-establishes. No turbulence.
		Opening door	Vigorously opening the door of the operating room, while visualizing horizontal airflow (see “Visualization of horizontal airflow”).	See “Visualization of horizontal airflow”. Opening the door does not influence airflow in the cabinet.
		Slow movements around cabinet	Walking past the cabinet slowly, while visualizing horizontal airflow (see “Visualization of horizontal airflow”).	See “Visualization of horizontal airflow”. Walking past the cabinet does not influence airflow in the cabinet.
		Fast movements around cabinet	Walking past the cabinet fast, while visualizing horizontal airflow (see “Visualization of horizontal airflow”).	See “Visualization of horizontal airflow”. If any disturbances, airflow should re-establish immediately. This test is useful to visualize influence of fast movements.
		Placing vials and flasks	Placing vials of several frequently used sizes and shapes at several places in the working field.	Airflow is unidirectional and moves around the vial. If airflow is disturbed by placing objects in the cabinet, it re-establishes immediately.
		Aseptic process simulation	Perform an aseptic process simulation while supplying smoke over the working field (e.g., reconstitute powder in a vial and inject this into an infusion bag) [16].	First air^2^ should never be blocked near a critical spot [16]. This test is useful to visualize the effect of an operator’s actions.
**Grade B/C Cleanroom**	At rest		Generating smoke 60 cm away from the air exhaust grille.	Smoke streams are efficiently removed from the cleanroom. No smoke clogs up near the exhaust grilles.
			Generating smoke 60 cm away from the air supply grilles.	Smoke streams are quickly dispersed into the cleanroom air. Immediately after smoke is supplied, the fog starts to diffuse [13].
			Generating smoke on at least 4 predetermined locations in the cleanroom.	Smoke streams are quickly dispersed into the cleanroom air. Immediately after smoke is supplied, the fog starts to diffuse [13].
			Passing smoke along large objects in the cleanroom, such as equipment carts and work benches (30 cm away from the object).	Smoke quickly disperses and no smoke clogs up near these objects.
			Passing smoke along imperfections in walls, ceiling and floor (30 cm away from the surface).	Imperfections in walls, ceiling, and floor do not hinder the airflow. Smoke quickly disperses and no smoke clogs up near these imperfections.
			Passing smoke 60 cm away along doors and hatches in the cleanroom.	Smoke quickly disperses and no smoke clogs up near the closed doors and hatches. No smoke leaves the cleanroom.
	In operation		Generate smoke 30 cm above the floor. Let an operator walk over the floor as they do during normal operations.	Smoke quickly disperses and no smoke clogs up near the operator.
			Opening a door or hatch. Supply smoke 60 cm away from the door or hatch.	No air from outside the cleanroom is drawn into the cleanroom. Generated smoke remains in the cleanroom. Normal airflow is restored within <5 s.
			Opening a door or hatch vigorously. Supply smoke about 60 cm away from the door or hatch.	No air from outside the cleanroom is drawn into the cleanroom. Normal airflow is restored within <5 s.
			Let an operator run through the cleanroom. Generate smoke around the operator, 60 cm away from the operator.	After fast movements of personnel, normal airflow is restored within <5 s.

^1^: This test is only relevant for a biosafety cabinet. ^2^: First air is particle-free air exiting from the HEPA filter in a unidirectional air stream [4].

## Data Availability

Data are contained within the article and Supplementary Materials.

## Supplementary Materials

The following supporting information can be downloaded at: https://www.mdpi.com/article/10.3390/pharmacy10050101/s1, Document S1: Questionnaire; Table S1: Overview of smoke machines; Video S1: Placing large objects in the LAF cabinet, Video S2: Walking near the LAF cabinet.
