# Supplementary material for: The Development and Implementation of Airflow Visualization Studies (“Smoke” Studies) as a Training Tool in Aseptic Hospital Compounding Facilities
Source: Pharmacy (Basel). 2022 Aug 23;10(5):101. doi: 10.3390/pharmacy10050101 (PMC9498447; doi:10.3390/pharmacy10050101)
Supplement: Supplementary file 1 [file pharmacy-10-00101-s001.zip › Document S1 Questionnaire.pdf]

Vragenlijst Nascholing Luchtpatroonvisualisaties
Questionnaire Training Airflow Visualizations

Vragenlijst vooraf – Questionnaire before training

Deel 1 – Part 1

1. Wat is uw functie? – *What is your profession?*
   - □ Apothekersassistent – *Pharmacy assistant*
   - □ Assistent KFZ – *Assistant Clinical Pharmaceutical Care*
   - □ Farmaceutisch Medewerker – *Pharmaceutical operator*

2. Hoe lang bent u een gekwalificeerd bereider? – *How long have you been a qualified compounder?*

----

3. Hoe veel bereidingen voert u gemiddeld per week uit? – *Hoe many times a week do you (on average) compound medications?*
   - □ Meer dan 5 bereidingen per week – *More than 5 times a week*
   - □ 4-5 bereidingen per week – *4-5 times a week*
   - □ 2-3 bereidingen per week – *2-3 times a week*
   - □ 1-2 bereidingen per week – *1-2 times a week*
   - □ Minder dan 1 bereiding per week - *less than 1 time per week*

4. Hoe vaak bereidt u in een downflow LAF-kast (zoals kast A3)? – *How frequently do you compound in a downflow LAF-cabinet?*
   - □ >2 keer per week - *>2 times a week*
   - □ 1-2 keer per week – *1-2 times a week*
   - □ 2-4 keer per maand – *2-4 times a month*
   - □ <2 keer per maand - *<2 times a month*
   - □ Nooit - *Never*

5. Hoe vaak bereidt u in een crossflow LAF-kast? – *How frequently do you compound in a crossflow LAF-cabinet?*
   - □ >2 keer per week - *>2 times a week*
   - □ 1-2 keer per week – *1-2 times a week*
   - □ 2-4 keer per maand – *2-4 times a month*
   - □ <2 keer per maand - *<2 times a month*
   - □ Nooit- *Never*

6. Hoe vaak bereidt u in een downflow veiligheidswerkbank (zoals in het borstcentrum/satellietapotheken/cytostatica)? - *How frequently do you compound in a downflow safety cabinet?*
   - □ >2 keer per week - *>2 times a week*
   - □ 1-2 keer per week – *1-2 times a week*
   - □ 2-4 keer per maand – *2-4 times a month*
   - □ <2 keer per maand - *<2 times a month*
   - □ Nooit – *Never*

7. Welke typen bereidingen voert u minstens 1 keer per maand uit? (meerdere antwoorden mogelijk) – *Which types of compounding do you execute at least once per month?*

- □ VTGM voorraad (aseptisch) – *Preparing medications of administration (aseptic) in stock*
- □ VTGM Individueel (aseptisch, in de cleanroom/op de satelliet) - *Preparing medications of administration (aseptic)for individual patients*
- □ VTGM in serie (aseptisch, bijv. piptazo/cefuroxim) - *Preparing medications of administration (aseptic) in series*
- □ Cytostatica - *Cytostatics*
- □ Steriele voorraadbereidingen (autoclaveren van eindverpakking) – *sterile preparations in stock*
- □ Niet-steriele bereidingen – *non-sterile preperations*

Deel 2

1. Waarom worden aseptische bereidingen uitgevoerd in een LAF-kast of Veiligheidswerkbank? – *Why is aseptic compounding executed in a LAF-cabinet of safety cabinet?*

2. Noem minimaal één verschil tussen een LAF-kast en Veiligheidswerkbank. – *Name at least one difference between a LAF-cabinet and (bio)safety cabinet.*

3. Teken in onderstaande vlakken een voorbeeld van een turbulente en unidirectionele luchtstroom m.b.v. pijltjes (de pijltjes geven de richting van de luchtstroom weer) – *Draw in the spaces below examples of turbulent and unidirectional airstreams using arrows.*

Turbulent - *turbulent*	Unidirectioneel - *unidirectional*

4. Hoe denkt u dat de luchtstromen in een downflow veiligheidswerkbank (zoals kast M1 in het borstcentrum) verlopen? Teken de luchtstromen met pijlen in onderstaande afbeelding vanaf het HEPA filter tot aan afzuiging in de luchtroosters. – *How do you think that the airstreams flow through a downflow safety cabinet? Draw the airstreams using arrows in the figure below, starting at the HEPA filter to the extraction air vents.*

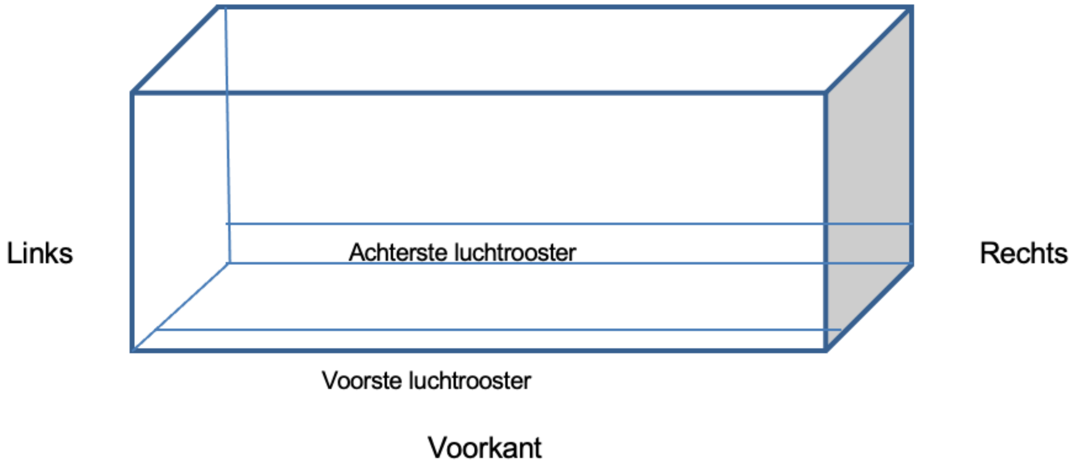

Translations:
Links = Left
Rechts = Right
Achterste luchtrooster = back vent
Voorste luchtrooster = front vent
Voorkant = front side

5. Hoe denkt u dat de luchtstromen in een crossflow LAF-kast) verlopen? Teken de luchtstromen met pijlen in onderstaande afbeelding vanaf het HEPA filter. *How do you think that the airstreams flow through a crossflow LAF-cabinet? Draw the airstreams using arrows in the figure below, starting at the HEPA filter.*

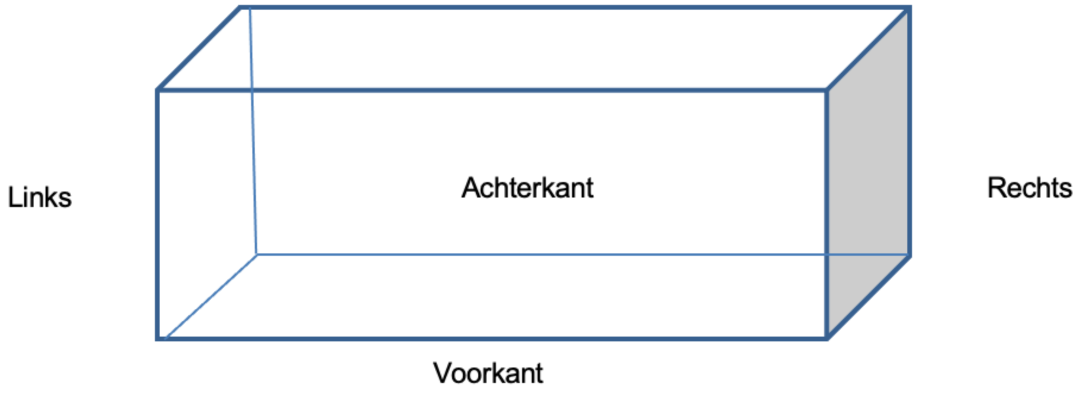

Translations:
Links = Left
Rechts = Right
Achterkant = back side
Voorkant = front side

6. U gaat een bereiding uitvoeren in een crossflow LAF-kast. U zit voor de LAF-kast. Waarom is een voorovergebogen houding hier ongewenst? (zie Figuur) – *You are compounding in a crossflow LAF-cabinet. Why is the leaned forward position (see Figure) undesirable?*

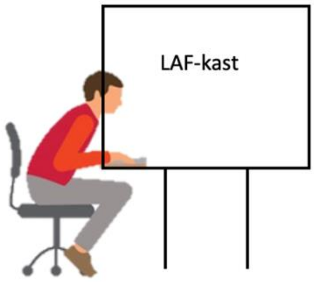

7. U gaat een bereiding uitvoeren in een downflow LAF-kast. Welke situatie zorgt voor een ongewenste blokkade van de luchtstroom? – *You are compounding in a downflow LAF-cabinet. Which situation results in blockage of the airstream?*

- □ De handen boven een (open) product houden – *Place hands above an (open) product*
- □ De handen voor een (open) product houden – *Place hands in front of an (open) product*
- □ De handen naast een (open) product houden – *Place hands next to an (open) product*
- □ De handen achter een (open) product houden – *Place hands behind an (open) product*

8. U gaat een bereiding uitvoeren in een crossflow LAF-kast. Aan welke opstelling geeft u de voorkeur? – *You are compounding in a crossflow LAF-cabinet. Which setup is preferred?*

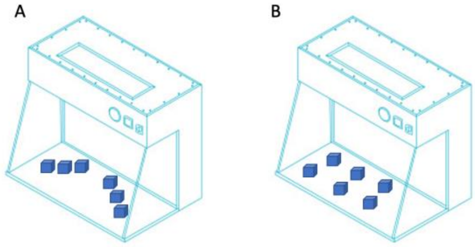

□ Opstelling A, omdat - *Setup A, because….*
□ Opstelling B, omdat - *Setup B, because….*

9. U gaat een bereiding uitvoeren in een downflow veiligheidswerkbank. Waarom is het ongewenst als u de voorwerpen dicht tegen elkaar plaatst? – *You are compounding in a downflow safety cabinet. Why should you not place objects close to eachother?*

10. U trekt 10 ml oplossing op uit een vial in een spuit m.b.v. een spike. U werkt in een downflow veiligheidswerkbank. Wat is de beste houding? – *U are taking 10 ml solution from a vial using a spike and a syringe. What is the best position?*

□ De vial en aangekoppelde spuit zo verticaal mogelijk houden, omdat – *Vial and syringe are helt vertically, because…*
□ De vial en de aangekoppelde spuit licht schuin houden, omdat – *Vial and syringe are helt slightly tilt, because…*

11. Denkt u dat luchtstromen vanuit de achtergrondruimte in een veiligheidswerkbank (zoals M1 in het borstcentrum) terechtkomen? - *Do you think that the airstreams from the background area enters the safety cabinet?*

□ Ja, lucht uit de achtergrondruimte komt de werkbank binnen. Ook nabij de zone waar wordt bereid. → Ga naar vraag 12 – *Yes, air from the background area enters the safety cabinet, even the area where compounding is executed. → Skip to question 12*
□ Ja, lucht uit de achtergrondruimte komt de werkbank binnen. Deze lucht komt niet verder dan de voorste luchtroosters → Ga naar vraag 12 - *Yes, air from the background area enters the safety cabinet, but does not pass the front vents → Skip to question 12*
□ Nee, geen enkele luchtstroom uit de achtergrondruimte komt de werkbank binnen. → Ga naar vraag 13 – *No, no airstreams from the background area enter the safety cabinet → Skip to question 13.*

12. U heeft hiervoor geantwoord dat lucht van de achtergrondruimte de veiligheidswerkbank kan bereiken. Tot hoeveel cm (gemeten vanaf het schuifraam van de veiligheidswerkbank) denkt u dat lucht van de achtergrondruimte de veiligheidswerkbank bereikt? – *You have answered that air from the background area enters the safety cabinet. Until how many cm (measured from the sash of the cabinet) do you think that the air from the background area enters the safety cabinet?*

_______________ cm

13. U plaatst een rookpistool 15 cm buiten een downflow veiligheidswerkbank om de luchtstromen te visualiseren. Teken hoe u denkt dat de luchtstromen, die de rook zichtbaar kan maken, verlopen. – *You are placing a smoke gun 15 cm outside a downflow safety cabinet to visualize airstreams. Draw in the figure below how you think that the airstreams flow.*

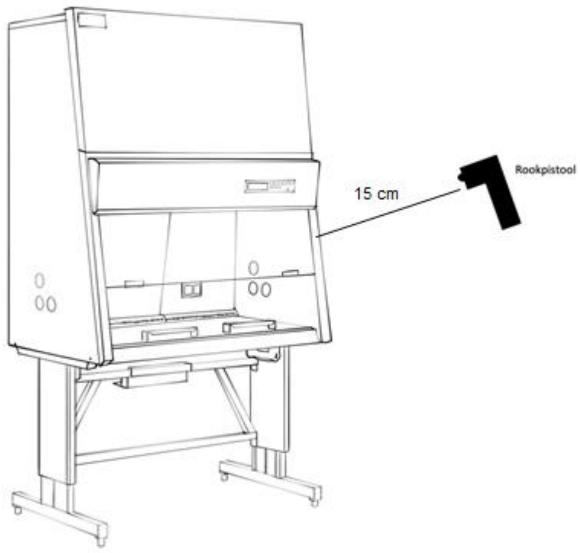

14. U voert een bereiding met een Baxa-pomp uit in een downflow-kast (zie Figuur). U pakt straks de aangekoppelde spuit en sluit deze af met een dopje. Heeft dit invloed op de luchtstroom? – *You are compounding using a Baxa-pump in a downflow cabinet (see Figure). You take a syringe and close it off with a lid. Does this influence the airstream?*

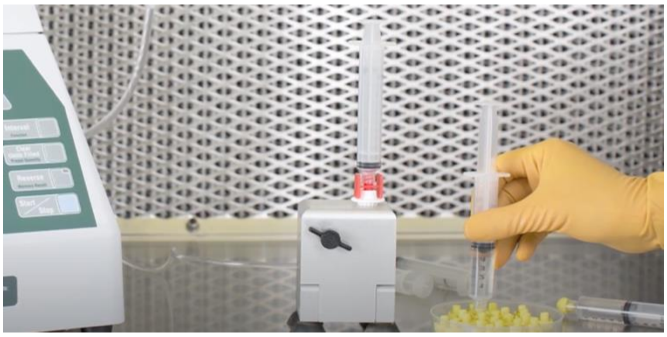

□ Ja, omdat – *Yes, because…*

□ Nee, omdat – *No, because..*

15. Denkt u dat het gebruik van een schudmachine (zoals de Orbital Shaker) invloed heeft op de (downflow) luchtstroom in een LAF-kast? – *Do you think that a shaker machine influences the downflow airstreams in a downflow cabinet?*
□ Ja, omdat – *Yes, because…*
□ Nee, omdat – *No, because…*

Deel 3

1. Houdt u rekening met de luchtstromen in de LAF-kasten of veiligheidswerkbanken tijdens het aseptisch bereiden? – *Do you hold the airstreams in LAF-cabinets or safety cabinets into account while compounding?*

- □ Ja, tijdens elke bereiding - *Yes, during every compounding session*
- □ Ja, maar ik vergeet dit af en toe wel - *Yes, but I do forget occasionally*
- □ Nee, ik houd geen rekening met de luchtstromen. – *No, I do not.*

2. Stelling: 'Het is belangrijk om rekening te houden met de luchtstromen in de LAF-kasten/veiligheidswerkbanken tijdens aseptische bereidingen'. – *Statement: 'It is important to hold the airstreams into account while compounding'*

- □ Helemaal mee eens – *Totally agree*
- □ Mee eens - *Agree*
- □ Een beetje mee eens – *Agree a little bit*
- □ Mee oneens – *Disagree*
- □ Helemaal mee oneens – *Totally disagree*

3. Verklaar uw voorgaande antwoord. Waarom vindt u het wel/niet belangrijk om rekening te houden met de luchtstromen in de werkbanken tijdens aseptische bereidingen? – *Explain your answer (question 2). Why do you feel it is /is nog important to hold the airstreams in the cabinets into account while compounding?*

4. Noem minimaal twee voorbeelden waaruit blijkt dat u rekening houdt met de luchtstromen in de LAF-kasten en veiligheidswerkbanken tijdens aseptisch bereiden. – *Name two examples that show that you take airstreams into account while compounding in LAF-cabinets or safety cabinets.*

5. Als u uzelf een cijfer zou geven voor uw aseptische technieken, wat zou dat dan zijn? – *What grade would you give yourself for your aseptic techniques?*

6. Kunt u een uitleg geven bij uw cijfer van uw aseptische technieken? Wat vindt u uw sterke punten? Wat vindt u eventuele verbeterpunten? – *Could you explain your grade? What are your strengths and weaknesses?*

Vragenlijst Nascholing Luchtpatroonvisualisaties
Questionnaire Training Airflow Visualizations

Vragenlijst achteraf – Questionnaire after training

Deel 1

- Identiek aan Vragenlijst vooraf – Same as Questionnaire before training

---

Deel 2
1. Stelling: 'Het is belangrijk om rekening te houden met de luchtstromen in de LAF-kasten/veiligheidswerkbanken tijdens aseptische bereidingen'. – *Statement: 'It is important to hold the airstreams into account while compounding'*

- □ Helemaal mee eens– *Totally agree*
- □ Mee eens - *Agree*
- □ Een beetje mee eens – *Agree a little bit*
- □ Mee oneens - *Disagree*
- □ Helemaal mee oneens – *Totally disagree*

2. Verklaar uw voorgaande antwoord. Waarom vindt u het wel/niet belangrijk om rekening te houden met de luchtstromen in de werkbanken tijdens aseptische bereidingen? – *Explain the answer of question 1.*

3. Als u uzelf een cijfer zou geven voor uw aseptische technieken (na afloop van deze nascholing), wat zou dat dan zijn? *What grade would you give yourself for your aseptic techniques (after training)?*

4. Zijn er factoren met betrekking tot het aseptisch bereiden die u nu anders zal gaan uitvoeren? (voorbeeld: plaatsing van vials, houdingen in de werkkast etc.) – *Are there any factors in regard to aseptic compounding that you would perform differently?*

5. Stelling: 'Rookvisualisaties zijn potentieel interessant om toe te passen tijdens de training van nieuwe bereiders' – *Statement: 'Smoke visualizations are potentially interesting to use during trainings of new compounders'*
- □ Helemaal mee eens – *Totally agree*
- □ Mee eens - *Agree*
- □ Een beetje mee eens – *Agree a little bit*
- □ Mee oneens - *Disagree*
- □ Helemaal mee oneens – *Totally disagree*

6. Kunt u uw antwoord op de voorgaande vraag toelichten? – *Could you explain your previous answer?*

7. Stelling: ‘Rookvisualisaties zijn potentieel interessant om toe te passen als nascholing voor gekwalificeerde bereiders’. – *Statement: ‘Smoke visualizations are potentially interesting to use during trainings of qualified compounders’*

- □ Helemaal mee eens – *Totally agree*
- □ Mee eens - *Agree*
- □ Een beetje mee eens – *Agree a little bit*
- □ Mee oneens - *Disagree*
- □ Helemaal mee oneens – *Totally disagree*

8. Kunt u uw antwoord op de voorgaande vraag toelichten? – *Could you explain your aswer?*

9. Welk cijfer geeft u de nascholing? – *What grade do you give this training?*

10. Wat vond u van de nascholing? Heeft u eventuele verbeterpunten? – *What did you think of the training? Do you have any feedback for us?*

11. Heeft u misschien aanbevelingen voor andere/nieuwe toepassingen voor rookstudies? Denk aan: te testen locaties, andere methodes, te gebruiken apparatuur etc. – *Do you have any recommendations for new applications of smoke studies?*
