# Supplementary material for: The Development and Implementation of Airflow Visualization Studies (“Smoke” Studies) as a Training Tool in Aseptic Hospital Compounding Facilities
Source: Pharmacy (Basel). 2022 Aug 23;10(5):101. doi: 10.3390/pharmacy10050101 (PMC9498447; doi:10.3390/pharmacy10050101)
Supplement: Supplementary file 1 [file pharmacy-10-00101-s001.zip › Table S1 Commercially available smoke machines.pdf]

Supplementary Material Table S1: commercially available smoke machines

Name Smoke Machine	Manufacturer	Machine Type	Smoke Type	Characteristics	Price	Website
**Flowmarker® / Flowmarker II™**	Tintschl	Portable	"SAFEX Fluid": alcohol, water and nitrogen preparation, MSDS available.	Wireless, attachables (hose and pipe with multiple outlets)	$ 5000	https://www.flowmarker.com
**FlowPointer®**	Tintschl	Smoke gun	SAFEX Fluid": alcohol, water and nitrogen preparation, MSDS available.	Wireless, 5-6 minute battery life, light smoke supply	On request	https://www.flowmarker.com
**Fog 2010**	Tintschl	Traditional	"SAFEX Fluid": alcohol, water and nitrogen preparation, MSDS available.	Dense fog, oil-free, attachable hose	On request	https://www.flowmarker.com
**°C Breeze**	Degree C	Portable	"Breeze Fog Fluid": Water, glycerol, glycol, MSDS available.	Wireless, attachables (various nozzles and pipe with multiple outlets)	$ 1200	https://degreec.com/pages/cbreeze
**Flow Check**	Dräger	Smoke gun	Preparation of propylene glycol,	10-minute battery life, light smoke supply, 3 minutes of	€ 500	https://www.draeger.com

			glycerol and water.	smoke generation per cartridge		
**Air Trace MK2 (S)**	Concept Smoke Systems	Portable	Unknown	Wireless, stainless steel option, adjustable smoke output, 15-20 minute battery life, attachable (pipe with multiple outlets)	€ 700	https://www.cmitest.com/produits/smoke-generator-air-trace/
**B1 Smoke Machine**	Concept Smoke Systems	Portable	Unknown	Very powerful portable machine, wireless, remote control, up to 10 minutes of operation	£ 650	https://www.cmitest.com/produits/b1-smoke-machine/
**Minicolt 4**	Concept Smoke Systems	Traditional	Glycerin-based	Wireless, stainless steel option, 0.2 µm diameter particles, 20-minute battery life.	€ 1000	https://www.cmitest.com/produits/minicolt-4/
**MFS Fog Generator**	Götsch Media	Traditional	Water for injections	Remote control, powerful device, various nozzles	€ 9000	https://www.mr-fogger.com
**Apollo.32**	Airflow Mappers	Traditional	Water for injections and liquid nitrogen	Attachables (various nozzles and pipe with multiple outlets), 10-12 m visible air flow distance	On request	https://airflowmappers.com/fog-generator-apollo32/
**AFM35-NEO AFM24-NEO**	Airflow Mappers	Traditional	Water for injections	Attachables (various nozzles and pipe with multiple outlets), 5-7 m visible air flow distance	On request	https://airflowmappers.com/afm-neo/

**CRF2 / CRF4**	Applied Physics USA	Traditional	Water for injections	50 minutes of use per water cycle, 8-10 µm droplets	On request	https://www.appliedphysicsusa.com
**Look Tiny S**	Look Solutions	Smoke gun	“Tiny Fluid”: alcohol and water preparation, MSDS available	Very powerful smoke gun, wireless, 10-minute battery life, low/high mode, disposable nozzle	€ 650	https://www.looksolutions.com
**AFM24**	Lighthouse (distributor)	Traditional	Water for injections	3-meter hose, spray lance to make mist curtains optional, fog intensity adjustable	On request	https://www.golighthouse.com/nl/contamination-control/ultrapure-cleanroom-fogger
